# Utilization of the Transtheoretical Model to Determine the Qualitative Impact of a Tribal FASD Prevention Program

**DOI:** 10.1177/2158244018822368

**Published:** 2019-01-14

**Authors:** Olivia Lowrey, Kaitlyn Ciampaglio, Jamie L. Messerli, Jessica D. Hanson

**Affiliations:** 1 Sanford Research, Sioux Falls, SD, USA

**Keywords:** alcohol-exposed pregnancies, American Indian health, alcohol consumption, birth control, transtheoretical model

## Abstract

Alcohol consumption during pregnancy can lead to damaging effects on an infant’s health, including fetal alcohol spectrum disorders. Project Changing High-risk alcOhol use and Increasing Contraception Effectiveness Study (CHOICES), a program developed to reduce alcohol-exposed pregnancies through decreased alcohol consumption and increased birth control use, has been implemented with success in a variety of populations. The CHOICES program was structured to align with the transtheoretical model (Stages of Change), a popular public health model. Although studies have described the Stages of Change in the context of a variety of health behaviors, none have addressed the qualitatively distinct characteristics of each stage in the context of American Indian (AI) women’s alcohol and birth control use. A framework analysis of 203 participants’ written responses during their experience in the Oglala Sioux Tribe (OST) CHOICES Program was conducted. As a conceptual framework, the transtheoretical model of behavior change was applied to the participants’ experiences, with two staff reading the open-ended responses and coding based on the stage of change. Participants’ responses suggest qualitatively distinct stages as well as a progression through the stages for both behaviors during the course of the program. Many participants mentioned their children, education, and work as inspiration to decrease their unhealthy behaviors. Common barriers to behavior change were found across both behaviors. The open-ended responses uncover common themes in the experiences of the participants. These results can help inform future programs which hope to address the needs of AI communities.

## Background

Fetal alcohol spectrum disorder (FASD) is the continuum of detrimental outcomes in individuals prenatally exposed to alcohol. This includes a diagnosis of fetal alcohol syndrome (FAS), which is evidenced by facial abnormalities, growth retardation, delayed brain grown, and neurobehavioral impairments (Hoyme et al., 2016). As FASD is caused by alcohol consumption during pregnancy, abstinence from alcohol when pregnant or when planning pregnancy, is highly recommended (American College of Obstetrics and Gynecologists, 2011; Centers for Disease Control and Prevention, 2005). Unfortunately, in the early months of pregnancy, many women are unaware that they are pregnant and continue to drink alcohol at risky levels (Floyd, Ebrahim, Boyle, & Gould, 1999).

FASD occurs in all socioeconomic and racial/ethnic groups. Recent research in an Upper Midwest study reported FAS in 5.9 to 10.2 per 1,000 children with no differences by race (May et al., 2014). Although drinking during pregnancy is higher among American Indian (AI) communities (May et al., 2004), surveillance rates of FASD in AI communities are sparse and not local to various tribal communities. However, when presented with opportunities to address FASD, AI communities have enthusiastically driven efforts to decrease the risk of an alcohol-exposed pregnancy (AEP), demonstrating a motivation and commitment to addressing this issue (Hanson & Pourier, 2015).

Although many FAS prevention programs focus on pregnant women, research suggests that a more effective approach places emphasis on decreasing alcohol consumption in women who are at risk for, or planning, pregnancy (Floyd et al., 2008). One preconceptual approach is Project *Changing High-risk alcOhol use and Increasing Contraception Effectiveness Study* (CHOICES), an evidence-based intervention that has demonstrated success in reducing risk for AEP (Floyd et al., 2007). Implemented with success in a variety of populations (Hanson et al., 2017; Letourneau et al., 2017; Velasquez, von Sternberg, & Parrish, 2013; Wilton et al., 2013), the CHOICES program focuses on either reducing binge drinking in women at risk for or planning pregnancy, and/or increasing usage of effective birth control to avoid unplanned pregnancies (Floyd et al., 1999; Floyd et al., 2007; Project CHOICES Intervention Research Group, 2003; Velasquez et al., 2010). The CHOICES program utilizes motivational interviewing (MI), a counseling style that “guides the individual to explore and resolve ambivalence about changing [behavior], highlighting and increasing perceived discrepancy between current behaviors and overall goals and values” (Project CHOICES Intervention Research Group, 2003, p. 1131).

The CHOICES Program was designed around the transtheoretical model (TTM), commonly known as the Stages of Change, which is particularly compatible with MI (Miller & Rollnick, 2002). TTM proposes that there are six stages that individuals go through while trying to change their behaviors (Prochaska, Redding, & Evers, 2008). Although in the *precontemplation stage*, individuals do not intend to change their behavior, either because they do not realize it is harmful, or they have failed to change in the past. In the *contemplation stage*, individuals understand that their behaviors have pros and cons, and they intend to change behaviors within the next 6 months. As individuals begin to develop plans toward their behavioral goal, they enter the *preparation stage*, where they intend to take action soon. The *action stage* is characterized by individuals who have made actual changes to their lifestyle to achieve their goal. Once the behavior change has been maintained for a significant period of time (usually defined as 6 months), the *maintenance stage* has been reached. Finally, in the *termination stage*, the individual has no temptation to return to their behavior and is confident that regardless of their circumstances, they will not relapse. MI, as used in the CHOICES Program, is particularly compatible with TTM as it allows for individualized plans that are tailored to a particular stage (Miller & Rollnick, 2002).

The purpose of this article is to describe movement through these Stages of Change for AI women enrolled in the Oglala Sioux Tribe (OST) CHOICES Program, a tribally run intervention where the original CHOICES program was modified to be more culturally relevant for implementation with AI women (Hanson & Pourier, 2015). Quantitative data from the OST CHOICES program demonstrated success in reducing risk for an AEP in AI women (Hanson et al., 2017); yet collective measures of outward behavior change fail to capture the perceptions and experiences of the women who participated in the program. This study adds a valuable qualitative approach to the TTM, which allows for a more dimensional understanding of the Stages of Change. It is distinct from previous research due to its combination of methods and content, as qualitative analysis of the TTM and focus on alcohol consumption, contraception use, and AI populations have not previously been studied in tandem. Among CHOICES research projects, this study presents an innovative way of reporting results, which is embedded within the Stages of Change, the framework that underlies the functionality of the CHOICES program itself. By using qualitative data from written responses that were collected during OST CHOICES sessions, distinct stages emerged and a more comprehensive picture was constructed, highlighting the common motivations and barriers to decreasing alcohol consumption and increasing contraception use.

## Method

Institutional review boards at the tribal level and at the principal investigator’s institution reviewed the program and found it did not constitute research but was instead a program evaluation that fell outside the prevue of the review boards. The OST CHOICES Program enrolled participants at three sites: two locations were on the reservation and a third in an urban setting approximately 2 hr from the reservation. Eligibility for the program was based on race (AI), age (18 years and older), and risky behavior status: Participants had to be drinking at binging levels—four or more drinks per occasion or eight or more drinks per week (U.S. Department of Health and Human Services, 2010). Eligibility also included women who were not pregnant but at risk for pregnancy: sexually active with a male, fertile, and not using any contraception during sexual intercourse or using a method incorrectly or inconsistently. Gift cards were provided as incentives to OST CHOICES participants. Demographic characteristics of our population are included in previous manuscripts (Hanson et al., 2017), with specific notes that all participants were nonpregnant, AI women who had binge drinking episodes in the past 90 days and were sexually active without using effective or any form of contraception.

### Measurements

OST CHOICES sessions were held across multiple locations and included two to four sessions approximately 1 to 2 weeks apart (Hanson et al., 2017). During the OST CHOICES sessions, MI counseling techniques were used by the program’s trained interventionists. The intervention employed several TTM-focused activities and open-ended questions. Specific activities in Session 1 included use of the Readiness Ruler, where participants were asked to rate, on a scale from 1 to 10, readiness to change behaviors, or to move from one stage to another. The Decisional Balance tool, derived from Janis and Mann’s (1977) model of decision making, was also used with participants to discuss the pros and cons of their current drinking and contraception behaviors. To reinforce the discussions on a participant’s level of willingness, readiness, and motivation to change her behavior during the first session, participants were asked to state their behavioral goals for both alcohol and contraception. Open-ended questions—called a *Change Plan*—were then utilized to help participants identify personal change goals for both alcohol and birth control.

During Session 2 or 4, the interventionist asked open-ended questions about any changes in alcohol and birth control use since the first session, and participants completed temptation and confidence scales regarding their temptation to use alcohol or not use birth control in specific situations, and alternatively, their confidence to not drink and/or to use birth control in those same situations. The temptation and confidence measures are particularly sensitive to the changes that are involved in progress in the Stages of Change and are good predictors of relapse (Plummer et al., 2001). After being asked to state their final goal statement for both alcohol and birth control, participants were asked to clarify their *Change Plan* regarding their alcohol and contraceptive use.

### Data Analysis

All the qualitative data responses were transcribed and analyzed via deductive content analysis (Elo & Kyngäs, 2008). A total of 203 qualitative responses were included for analysis. A codebook was developed using four of the six Stages of Change categories: precontemplation, contemplation, preparation, or action, as the time frame of the intervention did not allow for coding of maintenance or termination (see Table 1). Coders used common themes, phrases, and actions found in responses as indication in which stage of change participants appeared to be. The codebook went through an adaptation and development process involving multiple coding revisions, where two independent coders reviewed the codes and data to determine utility and consistency. When discrepancies between coders became apparent, the two coders and the principal investigator met to review. Coders then recoded all text to ensure consistency with the revised code-book (MacQueen, McLellan, Kay, & Milstein, 1998).

Inter-rater reliability was calculated using Cohen’s kappa. The two coders separately coded the responses using the final draft of the codebook. The Cohen’s kappa value was 0.58, “moderate” (0.41–0.60) when interpreted using the standards set by Landis and Koch (1977). Additional validity and reliability of the qualitative data were established by using verbatim responses, leaving little to interpretation. The results and discussion relate closely to the actual written responses of the participants and therefore are deemed to have strong validity.

## Results

### Precontemplation

#### Alcohol consumption.

Within the sample, relatively few women were in the precontemplation stage. Generally, women in the precontemplation stage did not see their drinking as problematic, or simply did not want to reduce their drinking. Many felt that their drinking levels could become problematic in the future, but that their current levels of drinking were acceptable. As one participant expressed, “It makes me think of how I can get into trouble when I do drink, but I don’t drink that much.” Alcohol consumption in certain scenarios was clearly understood to be connected to negative effects, with one woman stating, “I feel that my level of drinking is ok (but) if I did increase my drinking, it would increase the chance of getting in trouble.” Another woman did consider changing her drinking in the future, as “I don’t really think of nothing at the moment, but down the line eventually I do wanna stop drinking.”

#### Contraception.

Women in the precontemplation stage for birth control felt they did not need birth control, or did not express an interest to learn more about birth control. Within the sample, even fewer women were in the precontemplation stage for birth control use than they were for alcohol consumption. Although some women were worried about the side effects of birth control, many in the precontemplation stage simply stated that they did not want to use birth control and did not provide any reason.

### Contemplation

#### Alcohol consumption.

Many participants were seen in the contemplation phase, especially when it came to alcohol reduction. Logically this makes sense, considering that they were enrolled in a program dedicated specifically to addressing substance use. Women in the contemplation stage expressed a desire to decrease their drinking, and were aware that their drinking caused negative outcomes. Women were very cognizant of the effects drinking had on their family, and often named their family as one of the main reasons they wanted to decrease their drinking. One participant put it simply, stating, “I want to control my drinking, not my drinking controlling me.” Although women definitely desired to decrease their drinking, an overwhelming number of them lamented the fact that the activity of drinking is one that is socially embedded within many of their support systems, and was also a form of stress relief. Participants often reported that “stressful situations” and “peers I associate with, negative thoughts” were obstacles in their way of decreasing their drinking.

#### Contraception.

In the context of birth control use, the contemplation stage was manifested in women who expressed a desire to learn more about birth control, expressed the importance of birth control, stated that they did not want more children, or expressed a desire to use birth control more effectively. One participant was open in writing, “I am a risky drinker, and I don’t want any more children.” A large number of participants were similar; they felt that for a variety of reasons that it would be best to wait to have children. Many participants moved from contemplation to preparation from the first visit to the second. This is likely due to the emphasis on educating participants about contraception and on encouraging participants to make an appointment to see a health care provider.

### Preparation

#### Alcohol consumption.

In the preparation stage, women had decreased their drinking since their first CHOICES session, or had taken a step to decrease their drinking in the near future. Many of the women at this stage mentioned motivations to change such as their family, school, and jobs. For example, one woman expressed her reasons for decreasing her alcohol consumption were “because of my health, my kids, my freedom.” One challenge was difficulty of stopping drinking completely. Decreasing alcohol slowly over time was found to be a common solution, with one participant explaining, “Because it’s too hard to quit drinking all at once, so I’ll just try to slow down,” and another writing, “Take small steps at a time.” Women also mentioned that they felt the need to learn new ways to confront the conflicts that frequently provoked them to drink; one woman expressed that she wanted to learn “how to deal with conflict and temptation and people who are part of the problem.” Family and friends seemed to play an important part in this process, sometimes as the instigators: “My brothers come over and want to drink at my house every day. Told my brothers to stay away if they’re drinking.”

#### Contraception.

Women in the preparation stage had scheduled an appointment to meet with a health care provider, had independently learned more about birth control, or were using a birth control method, but inconsistently. Many women in this stage had appointments scheduled to begin birth control but expressed concern that they would be unable to attend their appointment due to other responsibilities or lack of transportation. Some women wanted to use condoms regularly as a form of birth control but felt worried that their partners would object to this. One woman stated she was “giving into the pressure of my boyfriend to not use condoms.” Another woman also mentioned that they would attempt to overcome this challenge by “talking with him about the importance of using condoms.”

### Action

#### Alcohol consumption.

In the context of alcohol consumption, the action stage was manifested in women who had changed their lifestyle to decrease their drinking, including attending Alcoholic Anonymous (AA) meetings or decreasing their drinking to below binge drinking levels. Barriers to continued sobriety included stress and temptation to drink socially. One woman expressed, “I am now tempted to drink when something bad happens or something good, but (I) cut down.” Many women were very focused on achieving goals to keep them on their chosen paths, for example, “I can keep track of my months of being sober.” Thinking about the consequences of their actions was also mentioned as a strategy to stay away from alcohol. One participant said that she would “think about it first and think about my baby first before drinking.”

#### Contraception.

For birth control use, women in the action stage had changed their lifestyle to avoid unplanned pregnancy by using a form of contraception. Many of the women in this stage expressed that they were not yet ready for children: “Not being ready for a baby is okay and don’t rush myself!” Many women also communicated an understanding that drinking while pregnant could cause damage to an unborn baby: “I’m currently on birth control and when I do decide to have my own child, he or she won’t be exposed to alcohol.” For some women, having medical appointments for a Depo shot and remembering to take the pill every day were concerns. One woman planned on “keepin’ sticky note on my mirror reminding me to take my pill.”

## Discussion

Quantitative data from OST CHOICES, as reported elsewhere, showed a significant decrease in AEP risk from baseline at both 3- and 6-month follow-ups, indicating the significant impact of the OST CHOICES intervention (Hanson et al., 2017). However, AI women in the OST CHOICES Program were more likely to reduce their risk for AEP by utilizing contraception. Binge drinking did decrease, but not at significant rates at 6 months postintervention and also not to below binge drinking rates. As readiness and motivation to make a behavioral change is a vital component of actual behavior change, qualitatively evaluating readiness via the TTM can help indicate where additional efforts are necessary within the OST CHOICES Program (Hesse, 2006; Joe, Simpson, & Broome, 1999; Prochaska, DiClemente, & Norcross, 1992).

The qualitative analysis indicates some of the reasons behind the success of the OST CHOICES intervention: that participants moved through the Stages of Change while participating in the intervention. At the first session, the majority of participants were in the contemplation stage for changing alcohol behavior, with few participants in the preparation stage and even fewer in the action stage. Most participants were likely in the contemplation stage specifically because of their inclusion into the OST CHOICES Program. At the second session, the distribution had changed, with less than half the previous number of participants in the contemplation stage, only a few participants in the preparation stage, and an increased number both in the preparation stage and in the action stage. As highlighted in the “Results” section, many participants had moved from thinking about change to actively making lifestyle changes to reduce risky drinking.

Similar results were found for contraception, although like the quantitative data results, contraception behaviors changed in a more striking way. At the first session, the majority of participants were in the contemplation stage for birth control, likely because of inclusion in the OST CHOICES Program, with few participants in the other stages. At the second session, the distribution had changed with many in the action stage (i.e., had made and sometimes even kept an appointment to be on birth control). We speculate this is because of the nature of the behavior change—making a birth control appointment and getting a prescription for birth control may be much more feasible to accomplish than decreasing alcohol consumption, especially when drinking is typically a social activity that many in a social network also participate in. For example, one woman explained changing her contraception was the most logical way for her to prevent FASD considering her personal situation: “I drink and I don’t want to have a FAS baby or bring someone else into this world because I can’t take care of it. So I got an IUD.”

This study is unique in several aspects. Few previous studies have utilized TTM within qualitative studies, and those that do, do not focus on the use of alcohol or contraception and do not involve AI populations. The one qualitative study that incorporated TTM with AI women worked to develop a culturally specific tool for mammogram screenings (Canales & Rakowski, 2006). TTM has been used extensively in alcohol reduction research, but mainly on a quantitative level (Campbell, 1997; Chang, McNamara, Wilkins-Haug, & Orav, 2007; Heather, McCambridge, Research, & Team, 2013; Hernandez-Avila, Burleson, & Kranzler, 1998; Hunter-Reel, McCrady, Hildebrandt, & Epstein, 2010; Reed et al., 2005; Velasquez, von Sternberg, Dodrill, Kan, & Parsons, 2005). In addition, several exploratory studies with wide ranges of participant demographics have used TTM as a framework for understanding and examining contraceptive use on a quantitative level (Choi & Shin, 2015; Emmett & Ferguson, 1999; Ha, Jayasuriya, & Owen, 2003, 2005). A number of studies looking to increase contraception use and initiation used TTM as a basis to develop an intervention, and then evaluate its effectiveness (Chung-Park, 2008; Davidson et al., 2015; Lee, Tsai, Tsou, & Chen, 2011; Peipert et al., 2008; Wang, Wang, Cheng, Hsu, & Lin, 2007).

Finally, our study is unique within the CHOICES literature, which has not yet had any results reported via qualitative means. As is noted in the introduction, the CHOICES intervention is based within the TTM. The CHOICES intervention employs several TTM methods, such as decisional balance and temptation/confidence exercises, which help to identify self-efficacy toward behavior change. However, previous CHOICES research has focused on the behavioral impact of the intervention, with no reported results on the process of change via the TTM lens nor qualitative data collected throughout the intervention (Floyd et al., 2007; Hanson, Ingersoll, & Pourier, 2015; Hanson et al., 2017; Letourneau et al., 2017; Project CHOICES Intervention Research Group, 2003; Wilton et al., 2013).

### Limitations

As noted from other OST CHOICES manuscripts, there were some limitations to our study. Our participants were typically self-referred, so the motivation to enter into the OST CHOICES Program may indicate that some women were already initiating behavioral change. In addition, all participants were AI women, and this sample was mainly women from one reservation community and one nontribal site, therefore results cannot be generalized.

## Conclusion

Overall, the results showed that women progressed through the course of the program. Specific activities such as decisional balance exercises, readiness rulers, and goal setting help to encourage women’s movement through the stages, and our qualitative data highlight some excellent evidence as to the “how” and “why” a participant moved through the stages. Several questions remain unanswered and can be addressed in future research. Although the program had a significant impact on contraception as shown in both the qualitative and quantitative results, more direct emphasis must somehow be placed on reducing risky drinking. The data, both qualitative and quantitative, show that CHOICES can work with AI/Alaska Native (AN) women, but additional efforts are needed for reducing alcohol use. Introducing specific interventions into the CHOICES intervention to address alcohol will take us to the next level in reducing risk for alcohol-exposed pregnancies with high-risk women.

## Figures and Tables

**Table 1.** Project Codebook.

Stage of change	Definition	Examples
Precontemplation	Alcohol: The woman does not see their drinking as problematic; does not want to change their behavior Birth control: The woman does not think they need birth control; expresses no interest in learning about birth control	“I feel that my level of drinking is ok, if I did increase my drinking, it would increase the chance of getting in trouble.” “I don’t want to be on birth control.”
Contemplation	Alcohol: The woman expresses a desire to decrease their drinking; is aware that their drinking is causing negative outcomes Birth control: The woman expresses a desire to learn more about birth control; thinks birth control is important; states that she does not want more children; expresses a desire to use birth control more effectively	“It’s hard on my kid when I drink, need to slow down.” “I can’t afford another kid, I’m not ready.”
Preparation	Alcohol: The woman has decreased drinking levels recently; has taken a step to decrease drinking in the near future Birth control: The woman has scheduled a doctor’s appointment; has independently learned more about birth control; uses a birth control method, but inconsistently	“Decreased drinking by 4 days … I want to better myself I’ll have more money or opportunities without drinking.” “I don’t see kids in the future … already made appointment. Birth control and being protected is a serious issue.”
Action	Alcohol: The woman has changed lifestyle to decrease drinking; has begun attending AA meetings; has decreased drinking below binge levels Birth control: The woman has changed lifestyle to avoid unplanned pregnancy; has begun effectively using a birth control method	“I haven’t drank since we last met and haven’t showed an interest in it.” “I discovered the Implanon and for me its more effective. I don’t have to remember it daily.”

Action *Note.* AA = Alcoholic Anonymous.

## References

[R1] American College of Obstetrics and Gynecologists. (2011). Committee opinion no. 496: At-risk drinking and alcohol dependence: Obstetric and gynecologic implications. Obstetrics and Gynecology, 118(2, Pt. 1), 383–388. 21775870 10.1097/AOG.0b013e31822c9906

[R2] Campbell WG (1997). Evaluation of a residential program using the Addiction Severity Index and stages of change. Journal of Addictive Diseases, 16, 27–39. 9083823 10.1300/J069v16n02_03

[R3] Canales MK, & Rakowski W (2006). Development of a culturally specific instrument for mammography screening: An example with American Indian women in Vermont. Journal of Nursing Measurement, 14, 99–115. 17086783 10.1891/jnm-v14i2a003

[R4] Centers for Disease Control and Prevention. (2005). Notice to readers: Surgeon General’s advisory on alcohol use in pregnancy. Morbidity & Mortality Weekly Report, 54, 229.

[R5] Chang G, McNamara T, Wilkins-Haug L, & Orav EJ (2007). Stages of change and prenatal alcohol use. Journal of Substance Abuse Treatment, 32, 105–109. 17175404 10.1016/j.jsat.2006.07.003

[R6] Choi MS, & Shin H (2015). Factors influencing stages of change for contraceptive use in college students: A path analysis. Nursing and Health, 3, 7–13.

[R7] Chung-Park MS (2008). Evaluation of a pregnancy prevention programme using the Contraceptive Behavior Change model. Journal of Advanced Nursing, 61, 81–91. 18173736 10.1111/j.1365-2648.2007.04468.x

[R8] Davidson AS, Whitaker AK, Martins SL, Hill B, Kuhn C, Hagbom-Ma C, & Gilliam M (2015). Impact of a theory-based video on initiation of long-acting reversible contraception after abortion. American Journal of Obstetrics Gynecology, 212(3), e1–e7. 10.1016/j.ajog.2014.09.027 25265403

[R9] Elo S, & Kyngäs H (2008). The qualitative content analysis process. Journal of Advanced Nursing, 62, 107–115. 18352969 10.1111/j.1365-2648.2007.04569.x

[R10] Emmett C, & Ferguson E (1999). Oral contraceptive pill use, decisional balance, risk perception and knowledge: An exploratory study. Journal of Reproductive and Infant Psychology, 17, 327–343.

[R11] Floyd RL, Ebrahim SH, Boyle CA, & Gould DW (1999). Observations from the CDC. Preventing alcohol-exposed pregnancies among women of childbearing age: The necessity of a preconceptional approach. Journal of Women’s Health and Gender-Based Medicine, 733–736. 10.1089/152460999319048 10495253

[R12] Floyd RL, Jack BW, Cefalo R, Atrash H, Mahoney J, Herron A, … Sokol RJ (2008). The clinical content of preconception care: Alcohol, tobacco, and illicit drug exposures. American Journal of Obstetrics Gynecology, 199(6 Suppl. 2), S333–339. 19081427 10.1016/j.ajog.2008.09.018

[R13] Floyd RL, Sobell M, Velasquez MM, Ingersoll KS, Nettleman MD, Sobell L, … Nagaraja J (2007). Preventing alcohol-exposed pregnancies: A randomized controlled trial. American Journal of Preventive Medicine, 32, 1–10. 17218187 10.1016/j.amepre.2006.08.028 PMC2888541

[R14] Ha BTT, Jayasuriya R, & Owen N (2003). Male involvement in family planning in rural Vietnam: An application of the transtheoretical model. Health Education Research, 18, 171–180. 12729176 10.1093/her/18.2.171

[R15] Ha BTT, Jayasuriya R, & Owen N (2005). Predictors of men’s acceptance of modern contraceptive practice: Study in rural Vietnam. Health Education & Behavior, 32, 738–750. 16267145 10.1177/1090198105277332

[R16] Hanson JD, Ingersoll K, & Pourier S (2015). Development and implementation of CHOICES group to reduce drinking, improve contraception, and prevent alcohol-exposed pregnancies in American Indian women. Journal of Substance Abuse Treatment, 59, 45–51. 26265591 10.1016/j.jsat.2015.07.006 PMC4661109

[R17] Hanson JD, Nelson ME, Jensen JL, Willman A, Jacobs-Knight J, & Ingersoll KS (2017). Impact of the CHOICES intervention in preventing alcohol-exposed pregnancies in American Indian women. Alcoholism: Clinical and Experimental Research, 41, 828–835. 10.1111/acer.13348 PMC5378586 28173632

[R18] Hanson JD, & Pourier S (2015). The Oglala Sioux Tribe CHOICES Program: Modifying an existing alcohol-exposed pregnancy intervention for use in an American Indian community. International Journal of Environmental Research and Public Health, 13, 1–10. 10.3390/ijerph13010001 PMC4730392 26703670

[R19] Heather N, McCambridge J & UKATT Research Team. (2013). Post-treatment stage of change predicts 12-month outcome of treatment for alcohol problems. Alcohol and Alcoholism, 48, 329–336. 23408241 10.1093/alcalc/agt006 PMC3633361

[R20] Hernandez-Avila CA, Burleson JA, & Kranzler HR (1998). Stage of change as a predictor of abstinence among alcohol-dependent subjects in pharmacotherapy trials. Substance Abuse, 19, 81–91. 12511809 10.1080/08897079809511377

[R21] Hesse M (2006). The Readiness Ruler as a measure of readiness to change poly-drug use in drug abusers. Harm Reduction Journal, 3, 3. 16436208 10.1186/1477-7517-3-3 PMC1395301

[R22] Hoyme HE, Kalberg WO, Elliott AJ, Blankenship J, Buckley D, Marais AS, … May PA (2016). Updated clinical guidelines for diagnosing fetal alcohol spectrum disorders. Pediatrics, 138, 1–18. 10.1542/peds.2015-4256 PMC4960726 27464676

[R23] Hunter-Reel D, McCrady BS, Hildebrandt T, & Epstein EE (2010). Indirect effect of social support for drinking on drinking outcomes: The role of motivation. Journal of Studies on Alcohol and Drugs, 71, 930–937. 20946752 10.15288/jsad.2010.71.930 PMC2965492

[R24] Janis IL, & Mann L (1977). Decision making: A psychological analysis of conflict, choice and commitment New York, NY: Free Press.

[R25] Joe GW, Simpson DD, & Broome KM (1999). Retention and patient engagement models for different treatment modalities in DATOS. Drug & Alcohol Dependence, 57, 113–125. 10617096 10.1016/s0376-8716(99)00088-5

[R26] Landis JR, & Koch GG (1977). The measurement of observer agreement for categorical data. Biometrics, 33, 159–174. 843571

[R27] Lee JT, Tsai JL, Tsou TS, & Chen MC (2011). Effectiveness of a theory-based postpartum sexual health education program on women’s contraceptive use: A randomized controlled trial. Contraception, 84, 48–56. 21664510 10.1016/j.contraception.2010.11.008

[R28] Letourneau B, Sobell LC, Sobell MB, Johnson K, Heinecke N, & Robinson SM (2017). Preventing alcohol-exposed pregnancies among Hispanic women. Journal of Ethnicity in Substance Abuse, 16, 109–121. 26727077 10.1080/15332640.2015.1093991

[R29] MacQueen KM, McLellan E, Kay K, & Milstein B (1998). Codebook development for team-based qualitative analysis. CAM Journal, 10, 31–36.

[R30] May PA, Baete A, Russo J, Elliott AJ, Blankenship J, Kalberg WO, … Hoyme HE (2014). Prevalence and characteristics of fetal alcohol spectrum disorders. Pediatrics, 134, 855–866. 25349310 10.1542/peds.2013-3319 PMC4210790

[R31] May PA, Gossage JP, White-Country M, Goodhart K, Decoteau S, Trujillo PM, … Hoyme HE (2004). Alcohol consumption and other maternal risk factors for fetal alcohol syndrome among three distinct samples of women before, during, and after pregnancy: The risk is relative. American Journal of Medical Genetics Part C: Seminars in Medical Genetics, C, 127, 10–20. 10.1002/ajmg.c.30011 15095467

[R32] Miller WR, & Rollnick S (2002). Motivational interviewing: Preparing people for change (2nd ed.). New York, NY: Guilford Press.

[R33] Peipert JF, Redding CA, Blume JD, Allsworth JE, Matteson KA, Lozowski F, … Rossi JS (2008). Tailored intervention to increase dual-contraceptive method use: A randomized trial to reduce unintended pregnancies and sexually transmitted infections. American Journal of Obstetrics & Gynecology, 198(6), e1–e8. 10.1016/j.ajog.2008.01.038 PMC2535847 18395692

[R34] Plummer BA, Velicer WF, Redding CA, Prochaska JO, Rossi JS, Pallonen UE, & Meier KS (2001). Stage of change, decisional balance, and temptations for smoking: Measurement and validation in a large, school-based population of adolescents. Addictive Behaviors, 26, 551–571. 11456077 10.1016/s0306-4603(00)00144-1

[R35] Prochaska JO, DiClemente CC, & Norcross JC (1992). In search of how people change: Applications to addictive behaviors. American Psychologist, 47, 1102–1114. 1329589 10.1037//0003-066x.47.9.1102

[R36] Prochaska JO, Redding CA, & Evers K (2008). The transtheoretical model and stages of change. In Glanz K, Lewis FM, & Rimer BK (Eds.), Health behavior and health education (4th ed., pp. 97–121). San Francisco, CA: Jossey-Bass.

[R37] Project CHOICES Intervention Research Group. (2003). Reducing the risk of alcohol-exposed pregnancies: A study of a motivational intervention in community settings. Pediatrics, 111, 1131–1135. 12728125

[R38] Reed DN, Wolf B, Barber KR, Kotlowski R, Montanez M, Saxe A, … Richardson JD (2005). The stages of change questionnaire as a predictor of trauma patients most likely to decrease alcohol use. Journal of the American College of Surgeons, 200, 179–185. 15664091 10.1016/j.jamcollsurg.2004.10.020

[R39] U.S. Department of Health and Human Services. (2010). Rethinking drinking: Alcohol and your health Washington, DC: NIH Publication.

[R40] Velasquez MM, Ingersoll KS, Sobell MB, Floyd RL, Sobell LC, & von Sternberg K (2010). A dual-focus motivational intervention to reduce the risk of alcohol-exposed pregnancy. Cognitive and Behavioral Practice, 17, 203–212. 20473352 10.1016/j.cbpra.2009.02.004 PMC2868197

[R41] Velasquez MM, von Sternberg K, Dodrill CL, Kan LY, & Parsons JT (2005). The transtheoretical model as a framework for developing substance abuse interventions. Journal of Addictions Nursing, 16, 31–40.

[R42] Velasquez MM, von Sternberg K, & Parrish DE (2013). CHOICES: An integrated behavioral intervention to prevent alcohol-exposed pregnancies among high-risk women in community settings. Social Work in Public Health, 28, 224–233. 23731416 10.1080/19371918.2013.759011

[R43] Wang RH, Wang HH, Cheng CP, Hsu HY, & Lin SY (2007). Testing a model of contraception use behavior among sexually active female adolescents in Taiwan. Research in Nursing & Health, 30, 628–640. 18022833 10.1002/nur.20222

[R44] Wilton G, Moberg DP, Van Stelle KR, Dold LL, Obmascher K, & Goodrich J (2013). A randomized trial comparing telephone versus in-person brief intervention to reduce the risk of an alcohol-exposed pregnancy. Journal of Substance Abuse Treatment, 45, 389–394. 23891460 10.1016/j.jsat.2013.06.006 PMC4055081

